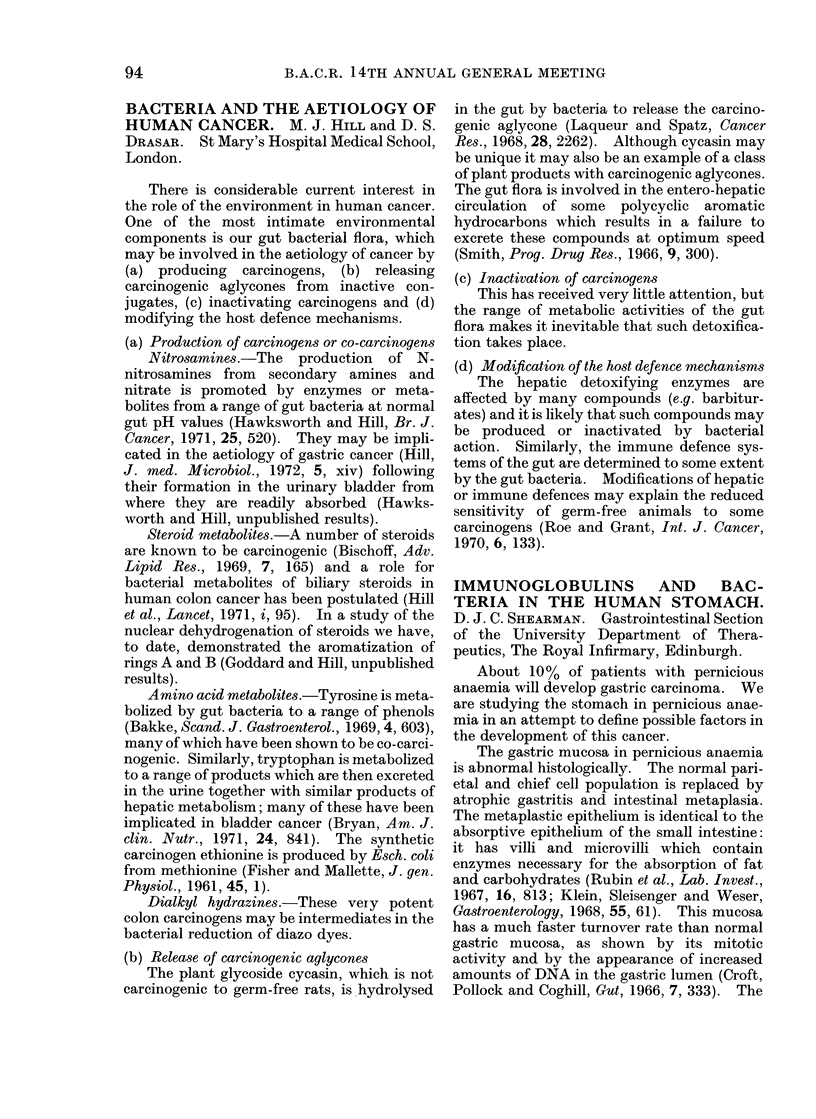# Bacteria and the aetiology of human cancer.

**DOI:** 10.1038/bjc.1973.122

**Published:** 1973-07

**Authors:** M. J. Hill, D. S. Drasar


### BACTERIA AND THE AETIOLOGY OF HUMAN CANCER.

M. J. Hill and D. S. Drasar. St Mary's Hospital Medical School, London.

There is considerable current interest in the role of the environment in human cancer. One of the most intimate environmental components is our gut bacterial flora, which may be involved in the aetiology of cancer by (a) producing carcinogens, (b) releasing carcinogenic aglycones from inactive conjugates, (c) inactivating carcinogens and (d) modifying the host defence mechanisms.

(a) *Production of carcinogens or co-carcinogens*

*Nitrosamines.*—The production of N-nitrosamines from secondary amines and nitrate is promoted by enzymes or metabolites from a range of gut bacteria at normal gut pH values (Hawksworth and Hill, *Br. J. Cancer*, 1971, **25**, 520). They may be implicated in the aetiology of gastric cancer (Hill, *J. med. Microbiol.*, 1972, **5**, xiv) following their formation in the urinary bladder from where they are readily absorbed (Hawksworth and Hill, unpublished results).

*Steroid metabolites.*—A number of steroids are known to be carcinogenic (Bischoff, *Adv. Lipid Res.*, 1969, **7**, 165) and a role for bacterial metabolites of biliary steroids in human colon cancer has been postulated (Hill *et al.*, *Lancet*, 1971, *i*, 95). In a study of the nuclear dehydrogenation of steroids we have, to date, demonstrated the aromatization of rings A and B (Goddard and Hill, unpublished results).

*Amino acid metabolites.*—Tyrosine is metabolized by gut bacteria to a range of phenols (Bakke, *Scand. J. Gastroenterol.*, 1969, **4**, 603), many of which have been shown to be co-carcinogenic. Similarly, tryptophan is metabolized to a range of products which are then excreted in the urine together with similar products of hepatic metabolism; many of these have been implicated in bladder cancer (Bryan, *Am. J. clin. Nutr.*, 1971, **24**, 841). The synthetic carcinogen ethionine is produced by *Esch. coli* from methionine (Fisher and Mallette, *J. gen. Physiol.*, 1961, **45**, 1).

*Dialkyl hydrazines.*—These very potent colon carcinogens may be intermediates in the bacterial reduction of diazo dyes.

(b) *Release of carcinogenic aglycones*

The plant glycoside cycasin, which is not carcinogenic to germ-free rats, is hydrolysed in the gut by bacteria to release the carcinogenic aglycone (Laqueur and Spatz, *Cancer Res.*, 1968, **28**, 2262). Although cycasin may be unique it may also be an example of a class of plant products with carcinogenic aglycones. The gut flora is involved in the entero-hepatic circulation of some polycyclic aromatic hydrocarbons which results in a failure to excrete these compounds at optimum speed (Smith, *Prog. Drug Res.*, 1966, **9**, 300).

(c) *Inactivation of carcinogens*

This has received very little attention, but the range of metabolic activities of the gut flora makes it inevitable that such detoxification takes place.

(d) *Modification of the host defence mechanisms*

The hepatic detoxifying enzymes are affected by many compounds (*e.g.* barbiturates) and it is likely that such compounds may be produced or inactivated by bacterial action. Similarly, the immune defence systems of the gut are determined to some extent by the gut bacteria. Modifications of hepatic or immune defences may explain the reduced sensitivity of germ-free animals to some carcinogens (Roe and Grant, *Int. J. Cancer*, 1970, **6**, 133).